# Body mass index is associated with lumbar disc degeneration in young Finnish males: subsample of Northern Finland birth cohort study 1986

**DOI:** 10.1186/1471-2474-14-87

**Published:** 2013-03-11

**Authors:** Jani Takatalo, Jaro Karppinen, Simo Taimela, Jaakko Niinimäki, Jaana Laitinen, Roberto Blanco Sequeiros, Markus Paananen, Jouko Remes, Simo Näyhä, Tuija Tammelin, Raija Korpelainen, Osmo Tervonen

**Affiliations:** 1 Institute of Clinical Medicine, Department of Physical and Rehabilitation Medicine, University of Oulu, PL 5000, Oulu, 90014, Finland; 2 Oulu University Hospital, Oulu, Finland; 3 Finnish Institute of Occupational Health, Health and Work Ability, and Disability Prevention Centre, Oulu, Finland; 4 Department of Public Health, University of Helsinki, Helsinki, Finland; 5 Institute of Diagnostics, University of Oulu, Oulu, Finland; 6 Institute of Health Sciences, University of Oulu, Oulu, Finland; 7 Center for Environmental and Respiratory Health Research, University of Oulu, Oulu, Finland; 8 LIKES Research Center for Sport and Health Sciences, Jyväskylä, Finland; 9 Department of Sports and Exercise Medicine, Oulu Deaconess Institute, Oulu, Finland

**Keywords:** Disc degeneration, Smoking, Body mass index, Physical activity, Waist circumference, Young adult

## Abstract

**Background:**

The role of environmental factors in lumbar intervertebral disc degeneration (DD) in young adults is largely unknown. Therefore, we investigated whether body mass index (BMI), smoking, and physical activity are associated with lumbar DD among young adults.

**Methods:**

The Oulu Back Study (OBS) is a subpopulation of the 1986 Northern Finland Birth Cohort (NFBC 1986) and it originally included 2,969 children. The OBS subjects received a postal questionnaire, and those who responded (N = 1,987) were invited to the physical examination. The participants (N = 874) were invited to lumbar MRI study. A total of 558 young adults (325 females and 233 males) underwent MRI that used a 1.5-T scanner at the mean age of 21. Each lumbar intervertebral disc was graded as normal (0), mildly (1), moderately (2), or severely (3) degenerated. We calculated a sum score of the lumbar DD, and analyzed the associations between environmental risk factors (smoking, physical activity and weight-related factors assessed at 16 and 19 years) and DD using ordinal logistic regression, the results being expressed as cumulative odds ratios (COR). All analyses were stratified by gender.

**Results:**

Of the 558 subjects, 256 (46%) had no DD, 117 (21%) had sum score of one, 93 (17%) sum score of two, and 92 (17%) sum score of three or higher. In the multivariate ordinal logistic regression model, BMI at 16 years (highest vs. lowest quartile) was associated with DD sum score among males (COR 2.35; 95% CI 1.19-4.65) but not among females (COR 1.29; 95% CI 0.72-2.32). Smoking of at least four pack-years was associated with DD among males, but not among females (COR 2.41; 95% CI 0.99-5.86 and 1.59; 95% 0.67-3.76, respectively). Self-reported physical activity was not associated with DD.

**Conclusions:**

High BMI at 16 years was associated with lumbar DD at 21 years among young males but not among females. High pack-years of smoking showed a comparable association in males, while physical activity had no association with DD in either gender. These results suggest that environmental factors are associated with DD among young males.

## Background

Failed nutrient supply to the disc cells has been claimed to be the primary cause of intervertebral disc degeneration (DD) [1]. The metabolism of the avascular intervertebral disc is dependent on the diffusion of fluid which solutes into and out of the disc, and the disc cells do not survive prolonged exposure to low glucose concentration [2,3]. Impaired nutrition could also explain the association between reduced lumbar artery blood flow and DD [4,5].

Environmental factors such as smoking, obesity and physical inactivity may enhance development of DD by reducing blood flow. In experimental models nicotine caused stenosis of vascular buds, perivascular calcification, necrotic changes in endothelial cells and narrowing of the vascular lumen [6] and smoking induced DD [7], obesity increases the risk of atherosclerosis due to related atherogenic dyslipidemia [8], and physical activity has a direct effect on the movement of nutrients into the disc [9].

The role of nutrition as the main initiator of DD has been questioned since genetic and early environmental factors accounted for most of the variance in DD, while environmental factors played only a negligible role [10,11]. However, almost all studies on the effect of environmental factors on DD have been performed in adult populations, and it is possible that the effect of environmental factors is more visible at a young age.

Our study population provides an excellent opportunity to assess the role of environmental factors in DD, as the cohort members have been followed up since birth. In the present study, we evaluated the effect of persistent smoking, body weight and physical activity at the ages of 16 and 19 years on lumbar DD in lumbar magnetic resonance imaging (MRI) performed at a mean age of 21.

## Methods

### Study population

**Figure 1:**
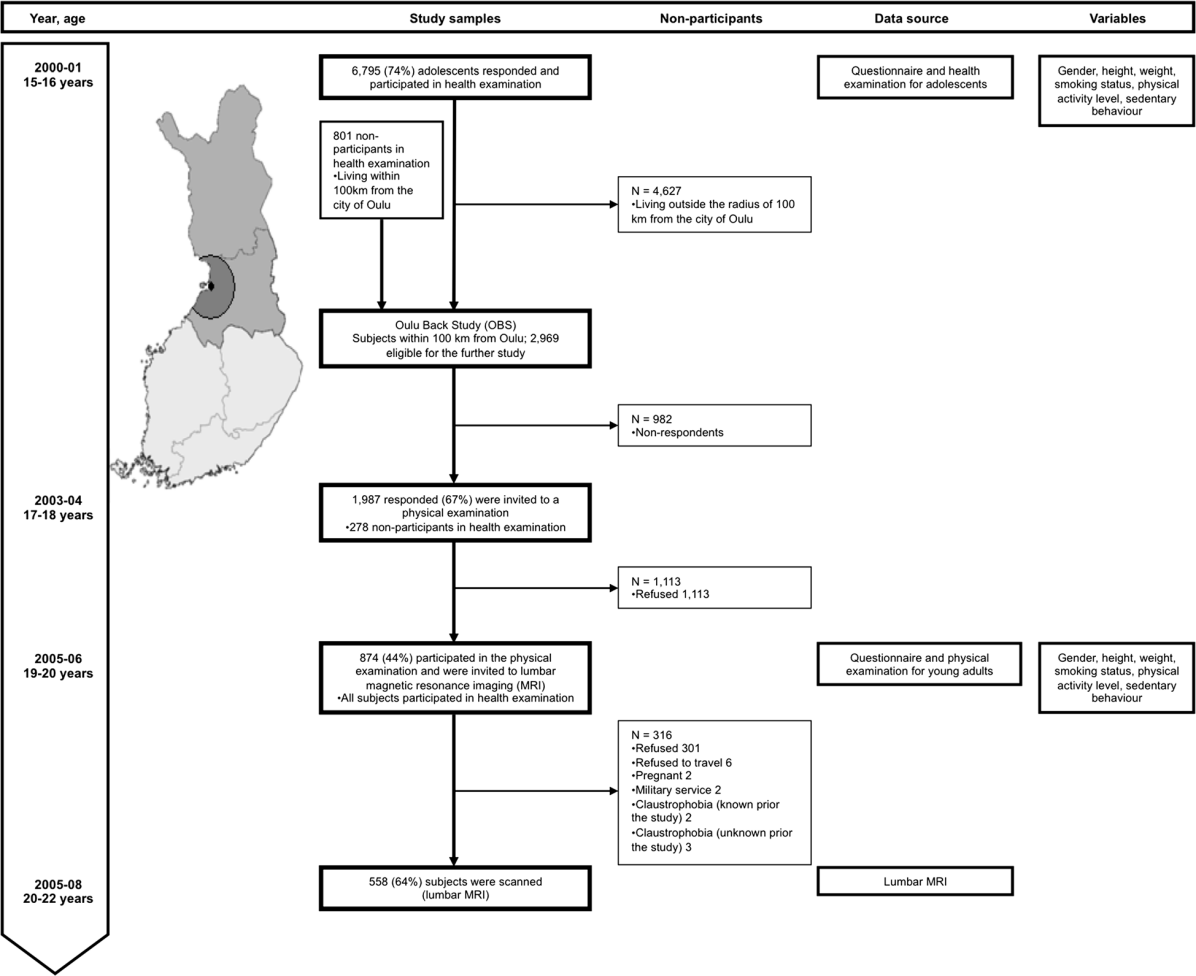
**Flow-chart of the study population.** The study population consisted of members of the 1986 Northern Finland Birth Cohort (NFBC 1986) in the two northernmost provinces of Finland (n = 9479). The study population comprised the 2969 subjects who lived within a radius of 100 km around of the city of Oulu in 2003. The participants of the physical examination were invited to lumbar magnetic resonance imaging (MRI), which was performed at the mean age of 21.

In 2001–2002, when they were approximately 16 years old, all living members of the 1986 Northern Finland Birth Cohort (NFBC 1986) whose addresses were known (n = 9215) received a postal questionnaire and 7344 adolescents (80%) responded. Questionnaire data, and body weight, height and waist circumference (WC) measured during the health examination at the age of 16 were available for 6795 subjects. In 2003–2004, when the cohort members were approximately 18 years old, a second postal questionnaire was sent to all those living within 100 km of the city of Oulu (n = 2969; Oulu Back Study; 801 subjects had not participated in the 16-year assessment). The respondents (n = 1987, 67% of those who received the questionnaire; 278 subjects had not participated in the 16-year assessment) were invited to a physical examination in 2005–2006 in which height, weight, and WC were measured. A total of 874 participants (44% of those invited, all having participated in the 16-year assessment) attended the examination at a mean age of 19. All participants of the physical examination were invited to undergo in lumbar spine MRI at a mean age of 21 (Figure 1). Of them, 563 attended MRI but five subjects interrupted the procedure due to claustrophobia. Therefore, a final total of 558 participants (64% of those who participated in the physical examination; 19% of the population of Oulu Back Study) completed MRI examination in 2007–2008.

 The study population was a subpopulation of the NFBC 1986, which consists of 9479 children with an expected date of birth between July 1, 1985 and June 30, 1986 in the two northernmost provinces of Finland; Oulu and Lapland.

 Some differences between the non-participants (n = 2408) and potential MRI participants (n = 563) have been previously reported [12]. In short, a slightly higher proportion of the participants were females (58% vs. 47%), physically more active (67% vs. 62% participating at least twice a week in brisk physical activity) and non-smokers (91% vs. 85%;) than non-participants, but a higher proportion of them suffered from low back pain (46% vs. 40%). We also noted that the non-participants had more missing data than the participants.

 The Ethics Committee of the Northern Ostrobothnia Hospital District approved the study plan and the study was performed according to the Declaration of Helsinki. The participants signed an informed consent prior the study enrollment.

### Lumbar magnetic resonance imaging

Participants were scanned using 1.5 T unit equipment (Signa, General Electric, Milwaukee, WI, USA) with a phased array spine coil (USA Instruments, Aurora OH, USA) and two imaging protocols of the entire lumbar spine: a sagittal T1-weighted (440/14 [repetition time msec/echo time msec]) spin echo, and T2-weighted (3960/116) fast spin echo. The slice thickness was 4 mm, with a 1 mm interslice gap. The detailed MRI protocol has been presented elsewhere [12].

 We used Modified Pfirrmann classifications (grades from one to five) to assess the degree of DD from T2-weighted images [12,13]. Grades 1 to 2 were classified as normal discs, while grades 3 to 5 were defined as degenerated. A sum score of DD was obtained by summing the scores of each lumbar disc. Normal discs (Grades 1 and 2) were scored as 0, and with each higher degree of DD the score increased by one. Therefore, the sum score could theoretically range from 0 to 15 for five lumbar discs, but the actual measurements yielded values of 0 to 8.

 DD was evaluated by two experienced musculoskeletal radiologists (JN and RB), who were blinded to all demographic and clinical data. The inter-rater reliability between the radiologists was poor for L1-2 and L2-3 disc degeneration (κ = 0.05 and 0.12, respectively), but moderate to good for the other levels (κ = 0.41, 0.63 and 0.50 for L3-4, L4-5 and L5-S1, respectively). Finally, all discrepancies were reviewed in consensus reading by the two radiologists.

### Anthropometric measures

Height and weight were measured at the physical examinations but also self-reported by the participants at 16 and 19 years of age. We calculated body mass index (BMI) as weight/height^2^ (kg/m^2^). Measured weight data was missing for eight subjects at 16 years and was replaced by the self-reported value. Waist circumference (WC) was measured halfway between the iliac crest and the lowest rib at 16 and 19 years. We calculated the difference of height, weight, BMI, and WC at both 16 and 19 years. No anthropometric data was available at 21 years.

### Level of leisure time physical activity

We evaluated the amount of physical activity outside school hours separately for moderate-to-vigorous and light physical activity by asking, ‘How many hours a week do you participate in a) brisk and b) light physical activity outside school hours?’ In the questionnaire the term brisk was defined as physical activity causing at least some sweating and getting out of breath (here referred to as moderate-to-vigorous intensity physical activity), and the term light as physical activity causing no sweating or shortage of breath. The response alternatives were: not at all, about half an hour, about one hour, 2–3 hours, about 4–6 hours and 7 hours or more. Moreover, the adolescents were asked about their daily time spent on physically active commuting to and from school [14]. The response alternatives (not at all, less than 20 min, 20–39 min, 40–59 min, and at least 1 hour per day) were multiplied by five (five school days a week) to correspond to 0, 1, 2.5, 3.75 and 5 hours per week. In calculations we used a metabolic equivalent (MET) intensity value of 3 for light physical activity, 5 METs for brisk physical activity and 4 METs for commuting physical activity [15]. Thus, 2.5 hours per week of each physical activity level corresponds to 30 MET hours per week (2.5h × 3 MET + 2.5h × 4 MET + 2.5h × 5 MET). We summed the MET hours per week at 16 and 19 years and calculated the mean value of physical activity for the analyses.

### Smoking

The number of pack-years of smoking was calculated by multiplying the number of packs of cigarettes smoked daily by years of smoking at the age of 19. Fifteen cigarettes were considered as one pack. Three groups of smoking status were formed: (1) non-smokers (reference category), (2) smokers with 0.01-3.99 pack-years of smoking, and (3) smokers with at least 4 pack-years of smoking.

### Statistics

The association of DD sum score (outcome) with the explanatory factors (anthropometric measures, physical activity and smoking) was examined by ordinal logistic regression based on proportional odds assumption. The outcome was the degree of DD, measured in ordered classes. The original ranks (0 to 8) were reclassified by combining the six highest classes (3 to 8) where the numbers were small, the final response variable having ordered values *i* = 1 to 4. The explanatory variables BMI, weight, height and WC were treated in quartiles to allow for curvilinear associations. The results were expressed as cumulative odds ratios (COR) and their 95% confidence intervals (CI). The COR expresses the ratio of odds for having a DD sum score greater or equal than that in any ordered class *i,* compared with that in all lower classes. This method combines the information from all ordered categories under the assumption that the odds ratios over all pairs of categories ≥ *i* vs. <*i* are similar. The proportionality assumption was checked by the score test using a 5% significance level [16]. Each explanatory variable was first entered alone (univariate analysis), followed by multiple regressions based on several variables. To avoid multicollinearity of anthropometric measures, only the strongest factors were retained in the final model. Analyses were stratified by gender. All analyses were performed using SAS software (version 9.1, SAS Institute Inc., Cary, NC, USA).

## Results

### Study population characteristics

Lumbar spine MRI was performed on 233 males and 325 females at a mean age of 21.2 years (range 20–23). Data on height, weight and BMI was available for 550 subjects at 16 years, waist circumference for 537 and 556 subjects at 16 and 19 years, respectively, and physical activity levels for 555 subjects, while pack-years of smoking, weight, height, and BMI at 19 years were available for all subjects. Table 1 shows the means and ranges for all anthropometric measures, MET hours per week, and the distribution of the subjects by pack-years of smoking.

**Table 1:** Means and ranges of anthropometric measures and physical activity level and the distributions of subjects by smoking class

	**Males**	**Females**	**Both sexes**	
	*Means (ranges) of anthropometric measures and physical activity*			*No. of valid observations*
BMI 16 yr (kg/m^2^)	21.2 (15.6–33.0)	20.7 (14.5–35.8)	20.9 (14.5–35.8)	550
BMI 19 yr (kg/m^2^)	23.8 (16.0–38.1)	22.1 (15.8–39.9)	22.8 (15.8–39.9)	558
Weight 16 yr (kg)	65.6 (45.2–112.1)	55.8 (37.5–94.0)	59.9 (37.5–112.1)	550
Weight 19 yr (kg)	75.7 (52.5–123.6)	59.8 (39.9–114.1)	66.4 (39.9–123.6)	558
Height 16 yr (cm)	175.6 (158.2–196.2)	164.1 (134.7–179.2)	168.9 (134.7–196.2)	550
Height 19 yr (cm)	178.3 (158.5-201.0)	164.3 (145.0-179.0)	170.1 (145.0-201.0)	558
Waist circumference 16 yr (cm)	75.9 (62.5-108.1)	70.4 (59.5-100.5)	72.7 (59.5-108.1)	537
Waist circumference 19 yr (cm)	81.8 (62.5-121.0)	72.3 (59.0-118.5)	76.3 (59.0-121.0)	556
Physical activity level (MET hours/week^#^)	34.6 (4.0-76.0)	29.6 (2.8-64.3)	31.7 (2.8-76.0)	555
	*Distributions of subjects by smoking class*			
Smoking (pack- years*) at 19 yr	No. (%)	No. (%)	No. (%)	
None	177 (76)	239 (74)	416 (75)	
0.01–3.99	37 (16)	67 (21)	104 (19)	
≥4.00	19 (8)	19 (6)	38 (7)	

^#^ Metabolic equivalent (MET) hours per week calculated from physical activity level outside school hours.

* Years of smoking multiplied by the packs of cigarettes smoked daily.

### MRI findings

**Figure 2:**
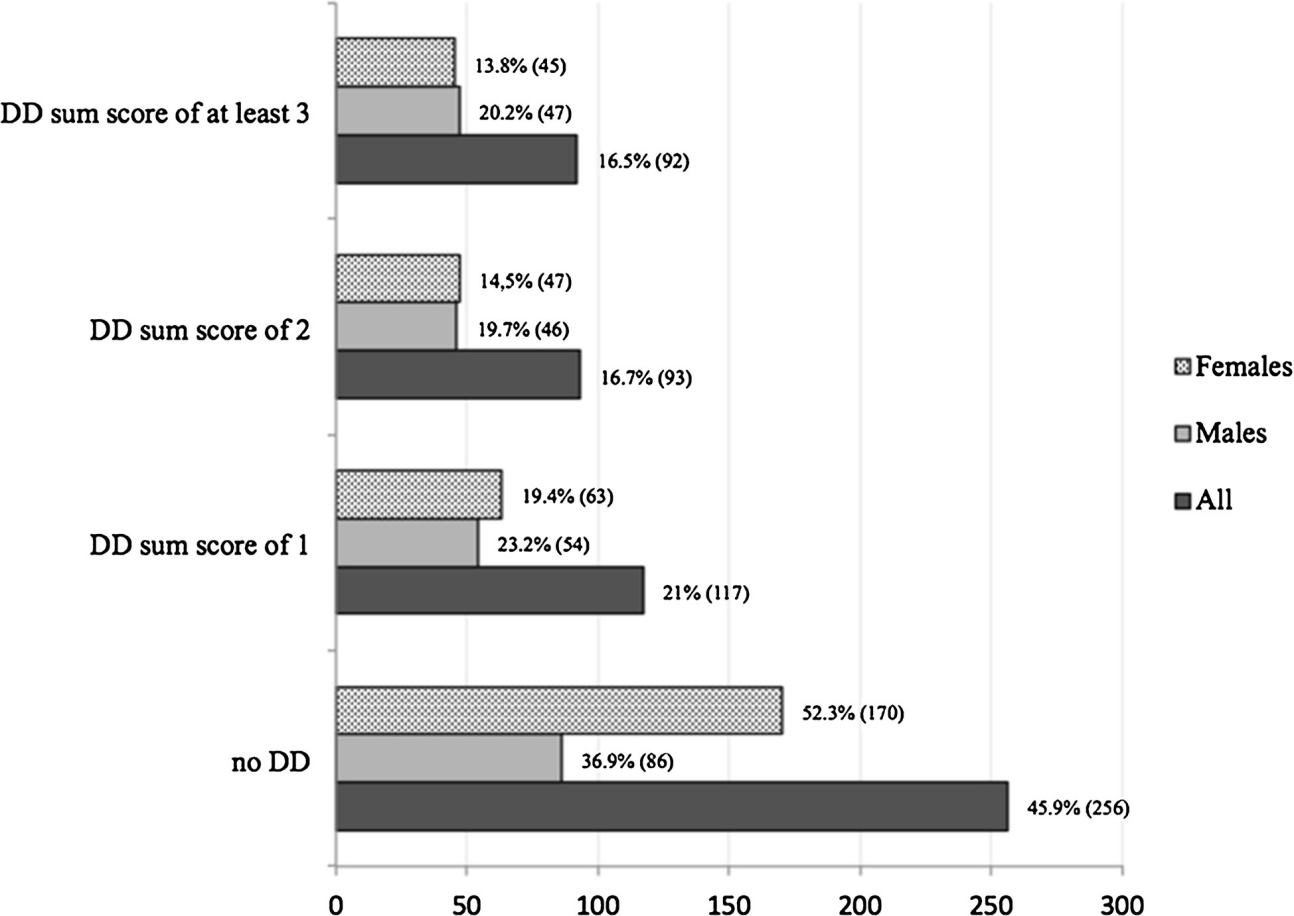
Prevalence of disc degeneration sum score of the lumbar spine in whole study population, males, and females.

Of the 558 subjects, 256 (46%) had no DD, 117 (21%) had sum score of one, 93 (17%) sum score of two, and 92 (16%) sum score of three or higher. The distributions were not very different in males and females otherwise but relatively more females than males (52% vs. 36%) had normal discs (Figure 2).

### Association of disc degeneration with environmental factors

The univariate analyses (Table 2) showed marked increases in COR by all anthropometric measures, from the lowest to highest quartiles, indicating an increase in the odds of belonging to any ordinal class of DD, or a class higher than that, compared with all lower classes. The increase of COR was mostly monotonic but not always linear. In males, the CORs in the highest quartiles ranged from 2.2 to 4.0, and their CIs remained well above the baseline, while in females, the CORs were much smaller, only height at 16 years exceeding the baseline with any certainty. All anthropometric measures showed higher CORs at 16 years than at 19 years. Male smokers with at least four pack-years showed a significantly high COR. Similarly in women, the COR was highest in heavy smokers, but the finding did not reach statistical significance. Physical activity was not associated with DD, although in females, the finding was suggestive of a U-shaped association with the lowest COR in the II quartile.

**Table 2:** Crude associations of lumbar disc degeneration sum score with quartiles of on body mass index (BMI), weight, height, waist circumference and physical activity level, and pack-years of smoking

**Males**		**Females**	
**(N = 230)**		**(N = 317)**	
*BMI at 16 years (kg/m*^*2*^*; quartiles)*			
I (15.6-19.0)	1.00	I (14.5-18.8)	1.00
II (19.1-20.6)	1.44 (0.74–2.81)	II (18.9-20.2)	0.72 (0.39–1.30)
III (20.7-22.6)	1.67 (0.85–3.26)	III (20.3-21.9)	1.04 (0.58–1.87)
IV (22.7-33.0)	2.64 (1.35–5.16)	IV (22.0-35.8)	1.33 (0.75–2.38)
*BMI at 19 years (kg/m*^*2*^*; quartiles)*			
I (16.0-21.1)	1.00	I (15.8-19.7)	1.00
II (21.2-23.1)	1.54 (0.80–2.99)	II (19.8-21.5)	0.93 (0.52–1.66)
III (23.2-25.6)	1.34 (0.69–2.61)	III (21.6-23.4)	0.83 (0.47–1.49)
IV (25.7-38.1)	2.22 (1.14–4.33)	IV (23.5-39.9)	1.09 (0.61–1.92)
*Weight at 16 years (kg; quartiles)*			
I (45.2-57.2)	1.00	I (37.5-49.9)	1.00
II (57.3-63.5)	2.04 (1.04–4.03)	II (50.0-54.0)	0.88 (0.48–1.61)
III (63.6-72.2)	2.18 (1.10–4.32)	III (54.1-60.3)	1.30 (0.72–2.34)
IV (72.3-112.1)	4.00 (2.01–7.96)	IV (60.4-94.0)	1.57 (0.88–2.81)
Weight *19 years (kg: quartiles)*			
I (52.5-66.9)	1.00	I (39.9-52.9)	1.00
II (67.0-73.4)	1.19 (0.61–2.32)	II (53.0-57.8)	1.24 (0.69–2.23)
III (73.5-81.5)	1.77 (0.91–3.44)	III (57.9-64.2)	1.23 (0.69–2.21)
IV (81.6-123.6)	2.72 (1.39–5.32)	IV (64.3-114.1)	1.42 (0.79–2.54)
*Height at 16 years (cm; quartiles)*			
I (158.2-171.3)	1.00	I (134.7-160.9)	1.00
II (171.4-174.9)	2.03 (1.02–4.04)	II (161.0-164.1)	1.28 (0.70–2.35)
III (175.0-179.8)	1.97 (1.01–3.84)	III (164.2-168.0)	1.68 (0.93–3.05)
IV (179.9-196.2)	3.39 (1.72–6.70)	IV (168.1-179.2)	1.81 (1.00–3.29)
*Height 19 years (cm; quartiles)*			
I (158.5-173.9)	1.00	I (145.0-160.9)	1.00
II (174.0-177.9)	1.01 (0.52–1.96)	II (161.0-164.4)	1.06 (0.58–1.93)
III (178.0-182.9)	1.31 (0.68–2.50)	III (164.5-167.9)	1.89 (1.04–3.42)
IV (183.0-201.0)	2.28 (1.16–4.46)	IV (168.0-179.0)	1.77 (0.98–3-18)
*Waist circumference at 16 years (cm; quartiles)*			
I (62.5-70.4)	1.00	I (59.5-65.9)	1.00
II (70.5-74.0)	1.02 (0.52–2.01)	II (66.0-69.0)	0.78 (0.43–1.42)
III (74.1-79.0)	1.67 (0.85–3.28)	III (69.1-73.4)	0.98 (0.54–1.78)
IV (79.1-108.1)	3.11 (1.58–6.15)	IV (73.5-100.5)	1.24 (0.69–2.22)
*Waist circumference at 19 years (cm; quartiles)*			
I (62.5-75.9)	1.00	I (59.0-66.9)	1.00
II (76.0-79.0)	1.29 (0.66–2.51)	II (67.0-70.0)	0.86 (0.48–1.52)
III (79.1-85.9)	1.50 (0.78–2.89)	III (70.1-75.0)	1.02 (0.57–1.85)
IV (86.0-121.0)	2.38 (1.23–4.62)	IV (75.1-118.5)	0.85 (0.47–1.53)
*Physical activity level*^*#*^*(MET hours/week; quartiles)*			
I (4.0-23.9)	1.00 (0.52–1.92)	I (2.8-20.0)	1.38 (0.77–2.47)
II (24.0-34.9)	1.00	II (20.1-28.9)	1.00
III (35.0-45.0)	1.16 (0.61–2.24)	III (29.0-39.0)	1.26 (0.70–2.27)
IV (45.1-76.0)	1.12 (0.58–2.16)	IV (39.1-64.3)	1.53 (0.85–2.74)
Smoking (pack-years*)			
None	1.00	None	1.00
0.01-3.99	1.49 (0.79–2.82)	0.01-3.99	1.09 (0.66–1.82)
≥4.00	2.53 (1.08–5.97)	≥4.00	1.64 (0.70–3.84)

^#^ Metabolic equivalent (MET) hours per week calculated from physical activity outside school hours.

* Years of smoking multiplied by the packs of cigarettes smoked daily.

Numbers are cumulative odds ratios relative to reference (95% confidence intervals).

 BMI at 16 years was selected to represent anthropometry in further analyses. The final model in Table 3 included the quartiles of BMI and physical activity, and pack-years of smoking. Compared with the univariate associations in Table 2, the CORs for BMI were slightly smaller and mostly exceeded the reference level in males but not in females. The association of smoking with DD in males attenuated after multivariate analysis to borderline statistical significance, but the confidence interval was wide and equally compatible with a 6-fold effect than no effect. The odds ratios for physical activity remained essentially unchanged. The score test indicated no violations of the proportional odds assumption at 5% level.

**Table 3:** Cumulative odds ratios (COR) and their 95% confidence intervals (CI) from multiple ordinal logistic regressions of the lumbar disk degeneration sum score (in four-class ordinal scale) on body mass index (BMI), physical activity per week and pack-years of smoking

**Explanatory factors**	**Males**	**Females**
	**N = 230**	**N = 317**
	COR (95% CI)	COR (95% CI)
BMI at 16 years (kg/m^2^; quartiles)		
I	1.00	1.00
II	1.44 (0.73–2.84)	0.67 (0.37–1.23)
III	1.64 (0.83–3.25)	0.99 (0.54–1.81)
IV	2.35 (1.19–4.65)	1.29 (0.72–2.32)
Physical activity level^#^ (MET hours/week; quartiles)		
I	1.00 (0.51–1.97)	1.38 (0.76–2.50)
II	1.00	1.00
III	1.27 (0.65–2.49)	1.27 (0.69–2.34)
IV	1.27 (0.65–2.50)	1.52 (0.84–2.75)
Smoking (pack-years*)		
None	1.00	1.00
0.01-3.99	1.47 (0.76–2.83)	1.09 (0.65–1.83)
≥4.00	2.41 (0.99–5.86)	1.59 (0.67–3.76)

^#^ Metabolic equivalent (MET) hours per week calculated from physical activity outside school hours; mean value at 16 and 19 years.

* Years of smoking multiplied by the packs of cigarettes smoked daily.

## Discussion

High BMI at 16 and 19 years associated with lumbar DD at 21 years among males belonging to the birth cohort. Having smoked at least four pack-years was also associated with lumbar DD among males, even though the finding did not reach statistical significance at 5% level. Such associations were not observed among females. The level of physical activity was not related with DD in either gender.

The first degenerative changes such as increased cell death, cleft and radial tear formation, and cracks in the endplates can be seen already at 11 years of age at the time when the discs start to become avascular as the vessels penetrating into the disc through the endplates are obliterated [17]. At molecular level, DD is characterized by loss of proteoglycans, which leads to desiccation [18,19]. Desiccation can be seen in MRI as the decreased signal intensity of the nucleus pulposus. MRI studies, in accordance with histological ones, have found a high prevalence of DD already in adolescence or early adulthood [11,19-21] as we have earlier observed in our study population [12].

We are aware of only two previous studies on the association of DD with environmental factors among adolescents or young adults [22,23]. Both studies were cross-sectional in design. The Japanese study [23], consisting of 308 university athletes and 70 non-athlete university students, observed that competitive baseball and swimming was associated with DD. The authors did not, however, evaluate the role of overweight or smoking. Among 13–20-year-old Southern Chinese subjects (n = 83), overweight or obesity based on Asian-modified BMI was significantly associated with the severity of lumbar DD, while smoking was not [24]. Unfortunately, their sample size was too small to allow gender stratification.

In the current study, BMI was associated with DD among males. We used two time points, 16 and 19 years, to study this association. Our results are consistent with a prospective study among Finnish male adults (n = 129) in which persistent overweight (BMI ≥25 kg/m2) at 25 and 40–45 years was associated with DD at L2/3 to L4/5 in MRI. Moreover, overweight at 25 years was associated with incident DD over a four-year period while overweight at 40–45 years was not [25]. Another longitudinal study [26] used the radiographic assessment of Dutch women’s osteophytes and narrowing of the disc space over a nine-year period (n = 742; age range 45–64 years at baseline). BMI was a predictor of DD only among those without DD at baseline. Cross-sectional designs have found associations between overweight and DD, both positive [23,24] and negative [27]. Furthermore, BMI may be a risk factor of specific phenotypes of DD such as spondylosis and Modic changes [28-31]. Interestingly, among weight-disconcordant twins, overweight was associated with lesser desiccation of the intervertebral discs suggested to be due to slow adaption to mechanical loading [32]. The results were disputed by the findings of a British twin study in which weight-related factors were associated with DD [33].

A high level of physical activity is beneficial for metabolic syndrome and cardiovascular health [34]. Yet a high level of physical activity, especially participation in power sports, appears to accelerate the development of DD [23,35-37]. Indeed, this topic remains controversial as some studies have found a positive association between heavy physical loading, either in occupation or sports, and DD, but negative findings have also been published [38-40]. The harmful effects of sports may be due to trauma, or excessive physical activity causing abnormal stress on the structural components of the disc with failure of the motion segment [41,42]. In the current study, the overall level of physical activity between the ages of 16 to 19 years was not associated with DD with any certainty. We evaluated overall level of physical activity as MET hours per week, which earlier studies have not done. METs take into account every physical activity level and therefore subjects who exercise actively even at a low level may have high MET scores. MET acts as a surrogate measure of overall physical activity level, not only participation in physical exercise and sports. We did not, however, evaluate the association between different sports and DD and therefore, we cannot rule out existence of risk sports with harmful effect on disc well-being.

 We found an association between smoking and DD among young adult males, which attenuated only slightly in the multivariate analysis. Previous population-based studies have found no such trend [24,38,43]. Among smoking-disconcordant adult twins, smoking explained only 2% of the variance in lumbar DD, which can be regarded as a marginal effect [10,44]. However, in experimental studies, smoking has indisputably caused degenerative changes in the intervertebral discs [6,7,45]. It may well be that the association between smoking and DD can be observed more easily in a young population. Interestingly, a recent meta-analysis found a stronger association between smoking and low back pain (LBP) among adolescents than adults [46].

In our study, BMI was significantly associated with DD among males, whereas physical activity was not. Overweight may cause or accelerate DD by a low-grade systemic inflammatory state caused by obesity [47,48]. We cannot exclude gene-environmental factor interactions such as those shown for obesity and the *COL9A3* gene [49]. The deleterious effect of overweight on the well-being of intervertebral discs is supported by a recent study of surgical tissue samples, which showed that overweight correlated with histological degenerative abnormalities [50].

The clinical relevance of DD is questioned by the fact that its prevalence is high among asymptomatic subjects [51,52]. However, increasing evidence has emerged that DD is associated with LBP both at a young age [12,20,22,52,53] and in adulthood [33,54-56]. Large prospective population-based cohorts are needed to evaluate whether weight control or smoking cessation protect from low back pain.

The strength of our study is the population-based birth cohort design, in which many environmental factors have been assessed prospectively from birth onwards. For this study, we used data regarding persistent smoking, BMI and physical activity at 16 and 19 years. Anthropometrics (weight, height, WC) were based on measured values in most cases. BMI was calculated using measured height and weight, but we did not use the published cut-off values of obesity and underweight for children and adolescents [57,58], because only a few subjects were outside the limits of the cut-offs. Moreover, using measurements at 16 and 19 years allowed us to evaluate the impact of persistence, in anthropometric measures. An additional strength of our study is the narrow age range, which enabled us to minimize the confounding effect of age.

The main limitation of our study is the cross-sectional design of imaging, which prevents us from drawing conclusions about temporal patterns between environmental factors and MRI findings due to the lack of sequential MRI scans. Therefore, the onset and progress of DD in the lumbar spine among the study subjects remains unknown. However, as DD has early onset [22], a very large cohort starting at early years with annual imaging for decades would be needed in order to study the natural progression of degenerative changes and their association with unhealthy behaviors.

A further limitation is that the data on smoking consisted of self-reported values. We used pack-years at 19 years and for physical activity the mean of MET hours per week at 16 and 19 years. We assume that young adults are more likely to underreport smoking than to overestimate it and, hence we regard the association between smoking and DD among males plausible. Assessment of physical activity was also based on self-reported values, which may have led to over-reporting of physical activity [59]. Unfortunately, more objective methods for assessment of physical activity such as pedometers, accelometers and heart rate monitors were not available in the present study. However, the test-retest reliability of physical activity questionnaire has been shown to be good at 16-year old population [14]. Correlation between self rated brisk physical activity and measured aerobic fitness has earlier been found to be acceptable also among 8 and 10 year old schoolchildren [60]. Another limitation of our study was that DD was evaluated visually from MRI. We used qualitative Pfirrmann classification [13], which is considered inferior to the quantitative assessment of DD [61,62]. We agree that visual evaluation is more robust than quantitative assessment but we have earlier found a moderate-to-good inter-rater reliability art the three lowest levels [12]. The kappa-values were lower than previously reported [63], but the disagreements were settled by consensus in our study. Moreover, due to the young age of our participants, the influence of environmental factors on lumbar DD might be less than genetic factors. However, associations of environmental factors with DD clearly existed among our study participants.

The MRI participants (n = 563) were more likely females, sat for shorter times, were more physically active, were more likely non-smokers and more likely suffered from LBP than non-participants to MRI of the original OBS postal survey (n = 2408). Thus, although some participation bias occurred, its impact on the generalizability of the results is likely to be small since the differences were minimal. Furthermore, no relevant bias was observed between the participants and nonparticipants among the subjects invited to MRI, as only BMI was found to be somewhat lower among the scanned subjects [12].

## Conclusion

Our study showed an association between BMI and DD among young males, whereas no such associations were observed among females. Also having smoked at least four pack-years showed a comparable association in males, which however failed to reach the 5% statistical significance. Physical activity had no significant association with DD in either gender. These results suggest that environmental factors are associated with DD among young males.

## Abbreviations

BMI: Body mass index; CI: Confidence interval; COR: Cumulative odds ratio; DD: Disc degeneration; LBP: Low back pain; MET: Metabolic equivalent; MRI: Magnetic resonance imaging; NFBC: Northern Finland birth cohort; WC: Waist circumference

## Competing interests

The author(s) declare that they have no competing interests.

## Authors’ contributions

JT read the lumbar MRIs, analyzed and interpreted data and had the most significant role in drafting manuscript. JK, ST, SN, JR and OT had significant role in study design and analyzing the data. JK and OT also enabled the collection of data. JN and RBS had significant role in interpreting the lumbar magnetic resonance images and had a contribution to data analysis. JL, TT and RK had significant role in interpreting the data. MP gave valuable advices on data in overall and helped in analyzing the data. All authors helped to draft the manuscript, and read and approved the final manuscript.

## Pre-publication history

The pre-publication history for this paper can be accessed here:

http://www.biomedcentral.com/1471-2474/14/87/prepub
